# Remote Photoplethysmography for Evaluation of Cutaneous Sensory Nerve Fiber Function †

**DOI:** 10.3390/s21041272

**Published:** 2021-02-11

**Authors:** Zbignevs Marcinkevics, Alise Aglinska, Uldis Rubins, Andris Grabovskis

**Affiliations:** 1 Department of Human and Animal Physiology, Faculty of Biology, University of Latvia, Jelgavas St.1, LV-1004 Riga, Latvia; 2 Biophotonics Laboratory, Institute of Atomic Physics and Spectroscopy, University of Latvia, Jelgavas St. 3, LV-1004 Riga, Latvia; aglinska96@gmail.com (A.A.); uldis.rubins@lu.lv (U.R.); andris.grabovskis@gmail.com (A.G.)

**Keywords:** cutaneous perfusion, remote photoplethysmography, chronic pain, neuropathic pain, pain assessment, cutaneous flowmotions, vasomotor response, topical heating

## Abstract

About 2% of the world’s population suffers from small nerve fiber dysfunction, neuropathy, which can result in severe pain. This condition is caused by damage to the small nerve fibers and its assessment is challenging, due to the lack of simple and objective diagnostic techniques. The present study aimed to develop a contactless photoplethysmography system using simple instrumentation, for objective and non-invasive assessment of small cutaneous sensory nerve fiber function. The approach is based on the use of contactless photoplethysmography for the characterization of skin flowmotions and topical heating evoked vasomotor responses. The feasibility of the technique was evaluated on volunteers (n = 14) using skin topical anesthesia, which is able to produce temporary alterations of cutaneous nerve fibers function. In the treated skin region in comparison to intact skin: neurogenic and endothelial component of flowmotions decreased by ~61% and 41%, the local heating evoked flare area decreased by ~44%, vasomotor response trend peak and nadir were substantially reduced. The results indicate for the potential of the remote photoplethysmography in the assessment of the cutaneous nerve fiber function. It is believed that in the future this technique could be used in the clinics as an affordable alternative to laser Doppler imaging technique.

## 1. Introduction

Small fiber peripheral neuropathy is a chronic nerve disorder, which occurs when damage to the peripheral nerves predominantly or entirely affects the small myelinated (Aδ) fibers or unmyelinated C fibers, which are involved in various somatic and autonomic functions [1], but mainly the sensory functions account for its manifestation such as thermal perception and nociception [2]. Therefore, the most debilitating symptoms of these impairments are ongoing burning pain, stabbing pain, squeezing or pressure pain, paroxysmal electric shock-like sensations, or mechanical dynamic allodynia [3], leading to a marked deterioration of the patients’ quality of life.

The exact prevalence of painful peripheral small fibers neuropathy is not known, as the disorder is associated with many diseases, including diabetes, infections, autoimmune and endocrine disorders, but it can also occur due to genetic alterations. In a review from 2015, Callaghan et al. reported that about 2–7% of the worldwide population might be affected by neuropathy [4] and this number in the near future may increase due to Coronavirus disease (COVID-19) neurological complications which affect both the central and peripheral nervous system [5,6,7].

Currently, the most common way to confirm small sensory nerve fiber damage is the quantitative somatosensory testing (QST) [8], which is a subjective and time-consuming method, or skin biopsy [9,10], which is invasive and uncomfortable for the patients. Among potential body sites suitable for diagnostics of chronic disease, the most promising is human skin, which is the largest organ, strategically located at the interface with the external environment with its complex diffuse neuro-immuno-endocrine system [11], which is able to represent the situation in the whole body [12,13], potentially serving as an early diagnostic marker [13]. In terms of the diagnostic side, the favorable feature of the skin is its immediate and complete accessibility for optic diagnostic techniques, partly due to superficial location, partly favorable optical properties, such as relatively large light penetration depth [14]. However, the challenging part is the fact that the structure of the skin is rather complicated [15], optical properties are not uniform [16] and may alter depending on the condition of regulatory mechanisms which are not fully elucidated. Thus, non-invasive assessment of cutaneous peripheral nerve fiber function is still challenging due to the current lack of simple and objective diagnostic techniques. Therefore, the development of objective methods is essential for diagnostics and disease course monitoring.

There are studies confirming the application of cutaneous microvascular bed for small nerve fiber function assessment, both during resting conditions using spectral analysis of blood perfusion oscillations (flowmotions) [17] and by examination of cutaneous perfusion in response to various provocation tests (reactivity tests) [18,19,20,21,22,23,24] which involve topical skin heating, topical skin cooling, application of mechanical compression and electrical stimuli [17]. This approach is based on the ability of cutaneous nerve fibers to mediate local vascular tone, which is manifested as blood perfusion changes. Despite various studies confirming the potential of skin for assessment of peripheral nervous system functions, such tests are still not used by clinicians as a routine procedure, which is partly due to the lack of reliability, specificity and sensitivity, partly due to the absence of simple and cost-effective instrumentation, the latter is supported by the fact that cutaneous perfusion has been extensively examined using Laser Doppler flowmetry technique, which is relatively expensive and therefore limited to a wide range of clinicians [25] including general practitioners. The cost-effective alternative could be remote photoplethysmography [26], which is a contactless optical technique for blood volume pulsation detection in the tissue using various computational algorithms [27] and relatively simple instrumentation: video camera, to detect subtle variations of back-reflected light, and appropriate light source. Currently, the imaging photoplethysmography (iPPG) is expanding its applications beyond the assessment of heart rate and arterial stiffness, illustrated by previous studies such as studies of Kamshilin et al. suggesting the high clinical potential of remote photoplethysmography in the evaluation of cutaneous vasomotor responses [27,28,29,30,31,32]. However, further refinement of this approach requires alteration of small sensory nerve fiber function in a controllable manner, which is difficult to achieve in neuropathic patients due to differences in etiology, degree of dysfunction, comorbidities, and other factors [33]. Therefore, the development of novel protocols mimicking peripheral nervous system dysfunction in healthy subjects for the assessment of optical techniques is of great importance.

The present study aimed to evaluate the feasibility of remote photoplethysmography for the assessment of small cutaneous sensory nerve fiber function on healthy young volunteers, using novel skin topical anesthesia protocol. It has been hypothesized that remote plethysmography can provide information related to cutaneous nerve fiber function.

## 2. Materials and Methods

### 2.1. Remote Photoplethysmography Setup

The major components of the developed photoplethysmography system were as follows: monochromatic camera USB-3.0, ADC 8–12-bits, resolution 648 × 488 pix. (Ximea MC023MG-SY-UB, XIMEA GmbH., Münster, Germany) with 540 nm–10 nm bandpass narrowband interference filter (Edmund Optics GmbH., Mainz, Germany), white industrial LED light source 50W (V-TAC, Shenzhen, China) and orthogonal polarizers film to reduce surface reflection. The choice of narrowband interference filter was based on our prior study, [30] confirming substantial improvement of signal quality as 540 nm corresponds to one of the Oxyhemoglobin absorption peaks.

**Figure 1:**
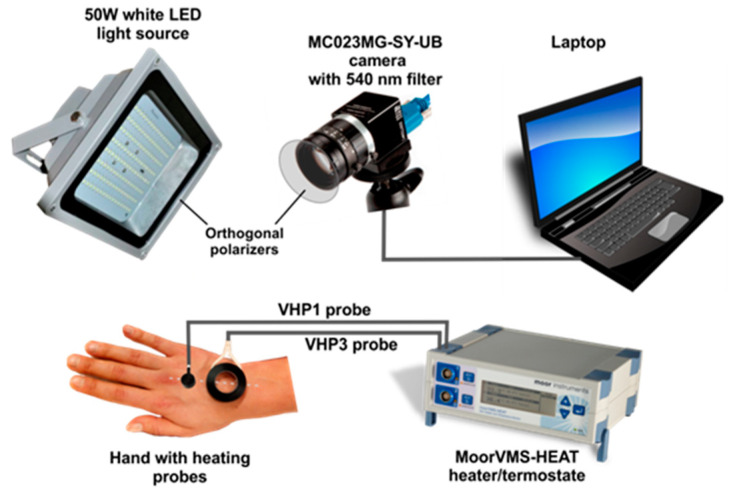
Remote photoplethysmography setup and used equipment; Palm dorsal aspect middle line is denoted by white dotted line.

Skin topical heating was performed using medical grade heater-thermostat (Moor VMS-HEAT, Moore Instruments, Ltd., Devon, UK) equipped with two heating probes (VHP1 and VHP3, Moore Instruments, Ltd., Devon, UK), and video data stored on the laptop computer (Dell Latitude 5510, Dell. Inc., Austin, TX, USA), see Figure 1. The video signal was acquired using a laptop computer operating Ximea software and stored to an uncompressed avi file.

### 2.2. Measurement Protocol

Fourteen (12 females and 2 males) clinically healthy volunteers (20–40 y) were recruited from the University of Latvia community. The study procedures were approved by the Ethics Committee of the University of Latvia, Institute of Cardiology and Regenerative Medicine (Prot.Nr: 03.05.2018), and was in accordance to the Declaration of Helsinki [34]. Prior to the study, all subjects were informed about the protocol and gave written informed consent. All measurements were performed at 21 ± 1 °C in a dark, well-ventilated room. The whole procedure comprised two consecutive protocols: *Induction of temporal cutaneous nerve fiber dysfunction* and *skin topical heating*. To simulate neuropathic alterations of small cutaneous sensory nerve fibers, skin topical anesthesia protocol described in Green et al., 2009 [24] was modified, so that control and anesthetized regions were situated close to each other on the same hand, with the advantage to acquire data from both sites simultaneously in one single frame using the same imaging system. The detailed description of our protocol is described below.

**Figure 2:**
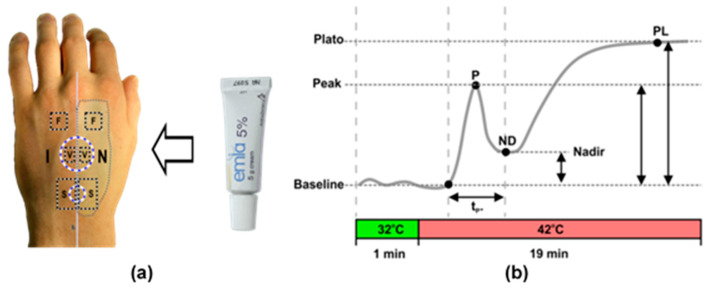
(**a**)-Position of probes and selected regions of interest following application of EMLA cream; I-intact skin region, N-EMLA gel numbed skin region. Position of the VHP3 and VHP1 probes are marked by large and small, blue dotted circles. Six regions of interest are indicated by black dotted squares: Flowmotion detection (F), vasomotor response trend (V) and flare area (S). (**b**)-Timeline of topical skin heating and typical trend of evoked vasomotor response; P-first peak, ND-nadir, PL-plato phase, tp-peak duration.

One hour before topical heating, the dorsal surface of the subject’s hand was divided into two regions by an imaginary middle line (Figure 1), on the left was the control region- with intact skin, and on the right was the region where an anesthetic substance (EMLA 5% Cream, AstraZeneca, Södertälje, Sweden) was carefully applied, so-called anesthetized region, Figure 2a. Following an hour exposure time, anesthetic cream was gently removed with 70% isopropyl alcohol whips (Alcohol Preps, Romed Inc., Wilnis, The Netherlands) skin was allowed to dry and the subject was asked to lie on the surgical chair (Mars 2, TRUMPF Medizin Systeme GmbH, Saalfeld, Germany) comfortably in a supine position with hand supported by vacuum pillow (AB Germa, Kristianstad, Sweden). To produce a topical heating induced response, the central point of VHP1 and VHP3 heating probes were placed on the imaginary line (midline), so that the same probe contacted anesthetized and intact skin regions. The VHP3 probe was attached to the skin using self-adhesive ring shaped tape and filled with distillated water, whereas VHP1 probe was gently placed on the skin and secured with the thin rubber belt as seen in Figure 1. Baseline perfusion was achieved by heating skin at 32 degrees for 1 min, while topical heating induced response was produced by heating skin at 42 degrees for 19 min. During the protocol, the video of the entire dorsal aspect of the hand was captured with monochromatic camera using manual exposure at 25 fps for 23 min, which later allowed selecting several separate regions of interest for offline analyses.

### 2.3. Signal Processing

Recorded videos were processed offline using custom designed MATLAB (Mathworks Inc., Novi, MI, USA) software (rPPG analyses, author: U. Rubins, University of Latvia, Riga, Latvia). The software performs computation of rPPG signals from recorded video file and calculates fast-varying (AC) and slow-varying (DC) rPPG components in order to detect dynamics of hemodynamic parameters, such as heart rate and blood volume changes in cutaneous tissue.

To compute skin repetitive low frequency (LF) oscillations, or so-called microvascular flowmotion which represent the influence of myogenic (0.05–0.15 Hz) [35], neurogenic (0.02–0.05 Hz) [36] and endothelial (0.0095–0.02 Hz) [37,38] activity on vascular tone, temporal component was filtered using 2nd order Butterworth zero-phase digital band-pass filter. To extract heartbeat-related fast-varying rPPG AC perfusion component, the signal was filtered in the same way in a frequency range 0.6–6 Hz.

During the analysis, the RoI is manually chosen over the skin region where the manipulations were done. The software calculates spatially averaged rPPG signal. Spatially averaged filtered rPPG signal can be found as:

(1) $$R_{n}=\frac{1}{M}\sum_{j=1}^{M}S_{j,n}$$

where *Rn* is spatially averaged signal *S_j,n_* obtained in *j*-th pixel in *n*-th video frame, in RoI containing *M* pixels. To obtain the set of perfusion parameters, the single-period waveform is calculated by beat-per-beat manner. The feet of waveforms are detected, by finding local minima. Then the set of perfusion index (*PI*) values is calculated in the following way:

(2) $$PI_{k}=100R_{k}/DC_{0,k}$$

where *PI_k_* is perfusion index, *DC*_0,*k*_ is zero-frequency DC signal, which are calculated in *k*-th beat of rPPG waveform. *PI(t)* signal was calculated in every heartbeat. To remove noise and outliers from *PI* signal, built in Matlab Hampel filter was used, functioning in the following way: for each sample of *PI*, the function computes the median of a window composed of the sample and its N surrounding samples. The standard deviation of each sample is estimated about its window median using the median absolute deviation. If a sample differs from the median by more than S standard deviations, it is replaced with the median value. For our measurements, we chose N = 30 and S = 0.1.

To obtain spatial distribution of blood perfusion in skin tissue perfusion map (AC or DC) was calculated using the *R_n_* as the ground truth reference signal by Pearson’s linear correlation coefficient matrix. The correlation was computed between the rPPG signal obtained in each pixel of image and the reference signal to determine the signal strength in each pixel of video:

(3) $$map_{j}=\frac{\sum_{n=1}^{N}\left(S_{j,n}-\bar{S_{j,n}}\right)\left(R_{n}-\bar{R_{n}}\right)}{\sqrt{\sum_{n=1}^{N}{\left(S_{j,n}-\bar{S_{j,n}}\right)}^{2}}\sqrt{\sum_{n=1}^{N}{\left(R_{n}-\bar{R_{n}}\right)}^{2}}}$$

where *n* is a frame count in video buffer, *S_j,n_* is a rPPG signal obtained in *j*-th pixel of *n*-th video frame, *R_n_* is the reference signal.

To compute cutaneous repetitive low frequency (LF) oscillations, the power spectrum density of band-pass filtered rPPG signal was calculated for three aforementioned spectral intervals using 16 min recording in 40 × 40 pixel RoI window. The power density was calculated in a following way:

(4) $$PD=\frac{1}{N}\sum_{n=1}^{N}Re{\left[F\left\{R\right\}\right]}_{n}$$

where *PD* is a power density, *Re*[*F{R*}] is a real part of Fourier transformed RoI-averaged signal, *N*-number of samples.

### 2.4. Analyses of Cutaneous Perfusion Data

The acquisition of the video from the entire dorsal aspect of the palm allowed selecting six regions of interest for further processing of cutaneous perfusion data. Characterization of vasomotor response trend was performed by selecting two regions inside VHP3 heating probe transparent part- one from intact skin, and one from anesthetized skin, and perfusion index changes over the time computed, Figure 2a. Four parameters were calculated from denoized perfusion index datasets: First peak amplitude (P), amplitude of nadir (ND), plato phase amplitude (PL) and first peak duration (tp) as depicted in Figure 2b. 

To explore flare response, two regions of interest surrounding VHP1 probe were selected (Figure 2a) and analyses performed on one-minute duration video fragment just following removal of the probe. Offline analyses included the generation of perfusion map, with the following determination of flare area in intact and anesthetized regions.

Cutaneous flowmotions were calculated from flare and heating unaffected skin, by selecting two ~40 × 40 pixel regions one from intact and the other from anesthetized skin in 16 min duration video fragment as depicted in Figure 2a. Flowmotions were divided into the three spectral ranges representing the influence of myogenic (~0.05–0.15 Hz) [35], neurogenic (~0.02–0.05 Hz) [36] and endothelial (~0.0095–0.02 Hz) [37,38] activity as suggested by other studies, and spectral power for each range was computed using Fast Fourier Transformas was described in the signal processing section.

### 2.5. Statistical Analyses

Statistical analyses were performed using SigmaPlot 12.0 (Systat Software Inc., San Jose, CA, USA). The data followed Gaussian distribution (Shapiro-Wilk test) therefore parametric statistical tests have been considered. The data of topical skin heating vasomotor response, and cutaneous flowmotions were analyzed using paired T-test and One-Way-Repeated Measure Analyses of Variance (One Way RM ANOVA), while the comparison of flare area in the control and EMLA treated regions were performed by paired T-test. Statistically significant difference is considered at *p* < 0.05. The values in the graphs are expressed as arithmetic mean ± standard error unless stated otherwise.

## 3. Results

### 3.1. Topical Heating Induced Flare Response

After removal of the VHP1 probe, the typical reddening of skin surrounding the probe (flare) was observed in all subjects, in both intact and anesthetized skin regions.

**Figure 3:**
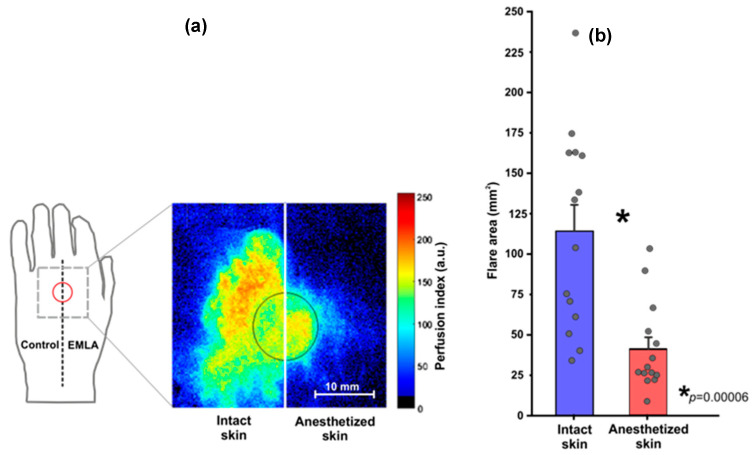
(**a**)-Perfusion index map exhibiting topical heating evoked flare response in intact and EMLA treated skin site- circle indicates position of VHP1 probe on the dorsal aspect of palm; typical example from one subject. (**b**)-Comparison of flare area in intact and EMLA treated skin region, group (n = 14) mean ± S.E.M., subjects individual measurements marked with black small circles. Statistically significant difference is denoted by asterisk.

Nevertheless, the significantly large flare area was observed in intact skin as shown in Figure 3a. The group mean flare area in intact skin region was 114.59 ± 59.03 mm^2^, and in the anesthetized region (EMLA treated region) was 41.30 ± 26.50 mm^2^ pointing out a substantial difference between intact and EMLA affected skin, with an average difference 73.30 ± 47.50 mm^2^ which correspond to 59.60 ± 17.90 percent changes, see Figure 3b.

### 3.2. Topical Heating Induced Vasomotor Response Trend

**Figure 4:**
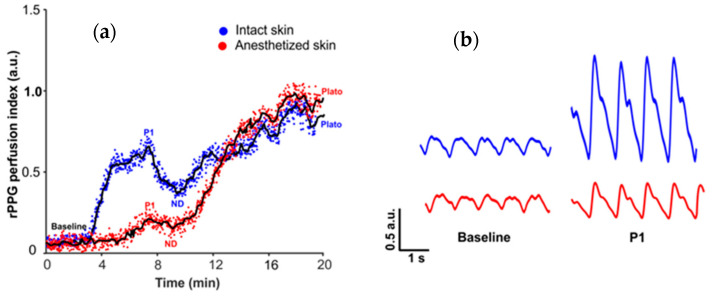
(**a**)-Topical skin heating (VHP3 probe) induced vasomotor response in intact and anesthetized skin; representative example from one subject, Characteristic parameters of vasomotor response are depicted on the graph: P1-first peak, ND-nadir. (**b**)-Example of photoplethysmography (rPPG) waveform during baseline and peak (P) from intact and EMLA treated skin (anesthetized skin); Example from one subject.

The typical vasomotor response, reported in other studies using laser Doppler flowmetry, was observed in the intact skin region during the skin topical heating test, comprising baseline perfusion during preheating, rapid increase of perfusion, the so-called first peak with the following nadir and relatively steady plato phase as depicted in Figure 4a.

While in the EMLA treated skin region, different vasomotor response was observed: The duration and amplitude of initial peak and nadir was substantially diminished, hence parameters of baseline perfusion and plato phase remained unchanged, (Figure 4a)-which is a noteworthy observation supporting the contribution of neurogenic mechanisms in formation of the initial peak.

**Figure 5:**
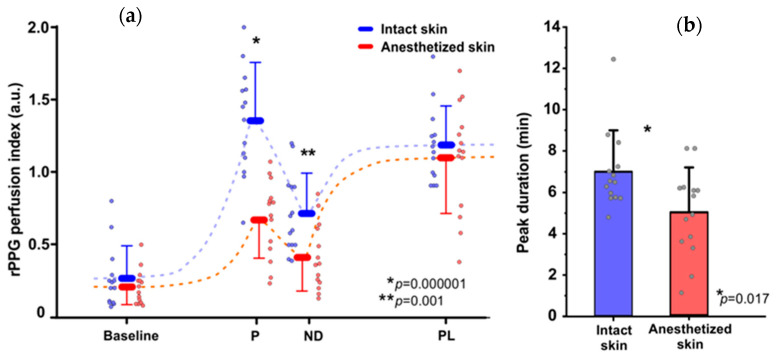
(**a**)-Topical skin heating induced vasomotor response in intact and anesthetized skin, group (n = 14) mean data ± S.E.M., subjects individual measurements marked with small circles; (**b**)-Topical skin heating induced vasomotor response peak duration, group (n = 14) mean data ± S.E.M. from intact and EMLA anesthetized skin regions, subjects individual measurements marked with black small circles. Statistically significant difference denoted by asterisks.

In the intact skin region the baseline perfusion was 0.27 ± 0.21 a.u., the initial peak amplitude reached 1.35 ± 0.36 a.u., while in EMLA treated region baseline perfusion was 0.21 ± 0.12 a.u. and initial peak amplitude was 0.68 ± 0.27 a.u. Similarly, in the intact region nadir amplitude was 0.71 ±0.27 a.u. but in anesthetized region 0.41 ± 0.23 a.u. as depicted in Figure 5a. Another observed effect of EMLA cream was an increase of initial peak duration: in intact skin duration was 6.99 ± 1.90 min, but in anesthetized region 4.99 ± 2.08 min, as shown in Figure 5b. The observed effects of EMLA gel on cutaneous blood perfusion during topical heating is mostly attributed to diminished amplitude of photoplethysmography AC component as depicted in Figure 4b, which largely determines perfusion index value.

### 3.3. Influence of EMLA on Cutaneous Flowmotions

**Figure 6:**
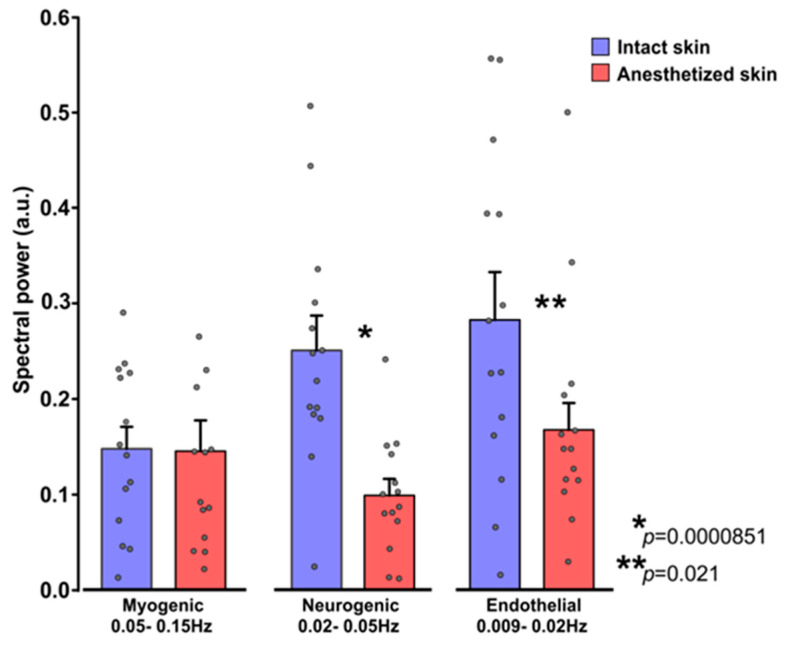
Cutaneous flowmotions obtained from intact and anesthetized skin regions, Group (n = 14) mean data ± S.E.M., small black circles depict subjects’ individual measurements; Statistically significant differences denoted by asterisks.

The average spectral power of flowmotions obtained from intact and EMLA treated skin region in three frequency ranges were presented in the bar chart in Figure 6. Unaffected skin exhibited relatively larger and statistically significant (One Way RM ANOVA; *p* = 0.001) power for endothelial (0.28 ± 0.17 a.u.) and neurogenic (0.25 ± 0.13 a.u.) frequency ranges in comparison to myogenic (0.15 ± 0.09 a.u.). 

Different spectral power values were observed in EMLA treated skin—the spectral power of neurogenic (0.10 ± 0.06 a.u.) and endothelial (0.17 ± 0.10 a.u.) frequency ranges were substantially lowered (neurogenic for ~61%, endothelial for ~41%) compared to intact skin region flowmotions. However, for the myogenic band (0.15 ± 0.12 a.u.), no difference was noted between intact and EMLA treated skin site.

Taken together, these results indicate that topical application EMLA gel to the skin significantly diminishes neurogenic and endothelial cutaneous flowmotions in remote photoplethysmography signal.

## 4. Discussion

The present study is a substantial extension of an earlier study presented at the conference materials [39] and its novelty is related to the development of protocol resembling derangement of small fiber function, similar to that observed in neuropathic patients. EMLA treated and intact skin regions were heated simultaneously with the same heating probe covering both regions, which allows us to capture different vasomotor responses using the same contactless photoplethysmography system, simultaneously, in very proximal cutaneous regions.

Whereas, in other studies using Laser Doppler techniques, either separate heater probes are placed on different limbs [40,41], or the measurement is performed from the same site but at different days- applying EMLA on one day and recording control the following day [42]. The detailed interpretation of our present findings is provided below.

### 4.1. Vasomotor Responses

The topical heating evoked vasomotor response obtained from intact skin region has a large similarity to that obtained in our earlier studies using contactless photoplethysmography technique [29], and laser Doppler registered response reported by other authors [43,44]. Laser Doppler studies suggest that during topical skin heating cutaneous hyperemia occurs [45], characterized by an initial peak within the first 5–6 min and a subsequent nadir followed by a sustained plateau [43]. Laser Doppler Imaging studies suggest that the peak is caused by an axon reflex, mediated by TRPV1 channel dependent activation of C-fiber afferent neurons that release substance P and calcitonin gene-related peptide (GCRP), with a modest contribution of NO [46] and may reflect both endothelial and small nerve fiber function [47,48]. That can explain the observation that EMLA treated skin exhibited a diminished initial peak, nadir and duration of initial peak, but not plato phase during the heat-induced vasomotor response. It is thought that the origin of plato phase is 60 to 70% NO-dependent, modulated by adenosine receptors, TRPV1 channels and reactive oxygen species [45]. A similar effect on initial peak duration was reported in other studies using Laser Doppler flowmetry, suggesting that topical application of lidocaine containing EMLA gel may change perfusion rise time [49,50]. The possibility of the direct influence of lidocaine as a major component of EMLA gel on cutaneous vasculature cannot be excluded, because of its vasodilator properties, possibly by the release of nitric oxide [51]. Nevertheless, this effect was not observed in our study using contactless photoplethysmography, as basal perfusion of EMLA anesthetized and intact skin regions did not substantially differ. The likely dilatory effect of lidocaine is masked by inhibition of cutaneous nerve fibers which dominate in regulation of cutaneous vascular tone. All subjects responded to EMLA gel and showed a similar trend of topical heating induced vasomotor response; however, the magnitude of response (time and amplitude of initial peak, nadir and plato phase) substantially differed across the subjects, possibly due to the inter-subject differences such as age, sex, and possibly vasoreactivity.

Another marker of cutaneous nerve fiber functions assessed in our study is the so-called vasomotor flare response, which manifests as the reddening of the skin outside the directly heated skin region. The evaluation of flare response as a parameter of vasomotor reactivity is widely used in different studies on cutaneous vasculature, using different provocation factors [48], such as Ach iontophoresis, electrical stimulation and topical skin heating; however, these studies mostly use Laser Doppler techniques (flowmetry or imaging) and cannot be directly attributed to photoplethysmographic data, as these techniques have different principles. The principle LDI is well defined, representing frequency change that light undergoes when reflected by red blood cells, thus output value is determined by flux-product of red blood cell linear velocity and concentration [52]. The same cannot be entirely said about PPG. The genesis of photoplethysmographic signal is not completely elucidated, hence modulation of PPG light intensity may be attributed to the mechanical movement of the capillary bed caused by varying arterial transmural pressure [53] and changes in RBCs orientation in the capillaries [54]. Nevertheless, studies suggest that PPG and LDI may share similar biophysics [55]. Our results confirm a decrease of flare area in EMLA treated skin, which is in line with other studies using Laser Doppler Imaging technique, suggesting a transitory suppressor effect of Lidocaine on the cutaneous sympathetic nerve fibers [56,57]. In the intact skin, topical heating depolarizes small unmyelinated dermal C fibers, which results in afferent action potentials that are conducted towards the spinal cord and at branching points antidromically activate peripheral branches, adjacent to the initial stimulation point [58]. This triggers the release of vasoactive substances, such as substance P and calcitonin gene-related peptide (CGRP), from nerve terminals and leads to a relaxation of arteriolar smooth muscle-vasodilation, resulting in increased perfusion at highly localized skin area, so-called flares [57]. While in anesthetized skin, these mechanisms are largely abolished leading to reduced flare area. Taken together these results confirm the role of neurogenic mechanisms in the manifestation of topical heating induced flare response, supporting other studies using laser Doppler flowmetry technique [20,22,40,59,60].

One issue observed in our data is large inter-subject variance of vasomotor responses, possibly caused by superimposition of several independent factors, such as a level of emotional stress, body composition, and phase of the menstrual cycle in female subjects or other confounding factors. Another issue is regarding comparison of our findings to those reported in other studies, which is rather difficult because of the diversity of protocols and used instrumentation, as the majority of existing studies used Laser Doppler Flowmetry or Laser Doppler Imaging techniques, which are different from photoplethysmography.

### 4.2. Cutaneous Flowmotions

Alongside provocation tests, which through selective stimulus can trigger blood perfusion response attributed to the particular physiological mechanism, another approach is the assessment of circulatory function in the baseline using so-called flowmotions [61]. Flowmotions are rhythmic oscillations in blood flow, to date widely assessed by Laser Doppler flowmetry, hence there are few studies reporting the use of contact manner reflection photoplethysmography [18,48,62]. The current exact genesis of these oscillations is unknown, but existing studies suggest the local origin as the dynamic interaction of sympathetic vasoconstriction, pressure-dependent vasoconstriction, flow-dependent endothelium-mediated vasodilation, metabolic vasodilation, and spontaneous myogenic activity [63].

The marked finding of the present study is the decrease of neurogenic and endothelial component in the EMLA treated skin, which is supported by Doppler studies on Diabetic patients with neuropathy [17]. Another interesting observation indicated the resistance of the myogenic component to EMLA treatment. Several other studies on neuropathic patients confirmed similar effects [17,63], thus we can speculate that EMLA treatment largely resembles dysfunction of cutaneous nerve fibers observed in neuropathic patients. However, to the best of our knowledge, there were no studies directly revealing the effect of EMLA gel on cutaneous flowmotions registered by photoplethysmography.

Summarizing, our results indicates that photoplethysmography derived cutaneous perfusion changes during vasomotor responses are similar to that reported by other studies using laser Doppler technique; however, the question remains whether the contactless modality of photoplethysmography could potentially substitute the gold standard techniques such as Laser Doppler imaging. Therefore, further studies should address this question.

### 4.3. Limitations of the Study

Despite the careful planning of the experimental design and the accurate implementation of all procedures, there are still several limitations that may potentially interfere with the present findings and therefore results should be interpreted with precaution.

The major limitation is related to the relatively small n = 14 and heterogeneous study group (20–40 y). On one hand, the selection of a small and heterogeneous group was dictated by the present situation with global Coronavirus pandemics. Therefore, meaning a minimal number of highly motivated local University staff were enrolled to follow strict epidemiological requirements. On the other hand, it was interesting to see whether observed effects are manifested in a relatively heterogeneous sample comprising subjects of different sex and age. We believe that the observed inter subject variability is related mostly to the subjects individual differences, such as: age [64], unknown status of former smokers [65], daily use of coffee and beverages [66], level of emotional stress during measurement procedure [67], degree of physical fitness [68] and unknown status of the menstrual cycle in female subjects [69]. Nevertheless, while the size of this preliminary study was not very large, it was sufficient to highlight statistically significant results in a largely heterogeneous group.

Another limitation is the lack of reference methods in the present study such as Laser Doppler, which is considered “the gold standard” for cutaneous vasomotor responses; therefore, it is difficult to compare the results with the other studies as they use Laser Doppler technique. There is ample evidence suggesting that cutaneous vasomotor responses registered simultaneously by both Laser Doppler technique and Photoplethysmography at 530 nm illumination have large similarities [28], which is also in line with other studies mentioning kinetics and pattern of topical heating induced vasomotor response obtained by laser Doppler [41,57,70], which corresponds to that obtained by PPG [30].

The question which remains largely unanswered in the present study is regarding the feasibility of contactless photoplethysmography in the assessment of neuropathic patients. This has not been directly addressed, as healthy volunteers instead of neuropathic patients were enrolled as test subjects. Also, instead of natural pathophysiology of nerve fiber derangement during the disease course, temporal alterations of intact nerve fibers were evoked using topical cutaneous anesthesia, namely EMLA gel. Whether these alterations are attributable if not similar to that of neuropathic patients is not entirely clear, as the mechanisms of neuropathic pathophysiology are not fully elucidated. Nevertheless, topical anesthesia protocol has been used in several studies using Laser Doppler [40], and authors concluded that the application of local anesthesia produced temporary alterations of cutaneous somatosensory function, as well as vasomotor response with a similar manifestation observed in neuropathic patients. Another confirmation comes from our prior pilot study on neuropathic patients [29], highlighting on the decreased flare response, which supports the capability of photoplethysmography for examining neuropathic patients. Therefore, we believe that the accumulated evidence indirectly confirms the potential of contactless photoplethysmography for the assessment of nerve fiber function in neuropathic patients. However, more studies are required on neuropathic patients using contactless photoplethysmography.

## 5. Conclusions

The present results indicate the potential of the remote photoplethysmography in the assessment of cutaneous small nerve fiber function as supported by EMLA anesthesia protocol. Therefore, it is believed that along with the existing gold standard techniques such as laser Doppler Imaging, the contactless modality of photoplethysmography in the future could be used for examination of neuropathic patients as a cost-effective and affordable alternative. However, more extensive studies are required on this subject.

## Data Availability

The data presented in this study are available in article Supplementary Files.

## Supplementary Materials

The following are available online at https://www.mdpi.com/1424-8220/21/4/1272/s1.
